# Mitochondria dysfunction in lung cancer-induced muscle wasting in C2C12 myotubes

**DOI:** 10.3389/fphys.2014.00503

**Published:** 2014-12-18

**Authors:** Julie B. McLean, Jennifer S. Moylan, Francisco H. Andrade

**Affiliations:** ^1^Department of Physiology, University of KentuckyLexington, KY, USA; ^2^Center for Muscle Biology, University of KentuckyLexington, KY, USA

**Keywords:** cachexia, mitochondria, oxidants, electron transport chain, skeletal muscle

## Abstract

**Aims:** Cancer cachexia is a syndrome which results in severe loss of muscle mass and marked fatigue. Conditioned media from cachexia-inducing cancer cells triggers metabolic dysfunction in skeletal muscle, including decreased mitochondrial respiration, which may contribute to fatigue. We hypothesized that Lewis lung carcinoma conditioned medium (LCM) would impair the mitochondrial electron transport chain (ETC) and increase production of reactive oxygen species, ultimately leading to decreased mitochondrial respiration. We incubated C2C12 myotubes with LCM for 30 min, 2, 4, 24 or 48 h. We measured protein content by western blot; oxidant production by 2′,7′-dichlorofluorescin diacetate (DCF), 4-amino-5-methylamino-2′,7′-difluorofluorescein diacetate (DAF), and MitoSox; cytochrome c oxidase activity by oxidation of cytochrome c substrate; and oxygen consumption rate (OCR) of intact myotubes by Seahorse XF Analyzer. Results: LCM treatment for 2 or 24 h decreased basal OCR and ATP-related OCR, but did not alter the content of mitochondrial complexes I, III, IV and V. LCM treatment caused a transient rise in reactive oxygen species (ROS). In particular, mitochondrial superoxide (MitoSOX) was elevated at 2 h. 4-Hydroxynonenal, a marker of oxidative stress, was elevated in both cytosolic and mitochondrial fractions of cell lysates after LCM treatment. Conclusion: These data show that lung cancer-conditioned media alters electron flow in the ETC and increases mitochondrial ROS production, both of which may ultimately impair aerobic metabolism and decrease muscle endurance.

## Introduction

Cancer kills nearly 600,000 Americans annually, equating to approximately 1500 deaths every day (Society, 2012). Fifty percent of all cancer patients experience cachexia, a severe wasting syndrome that includes loss of muscle mass, weakness and fatigue (Tan and Fearon, 2008; Fearon et al., 2011). Cachexia is unresponsive to nutritional interventions, and limits the response to cancer treatments (Fearon et al., 2013). Eighty percent of patients with advanced lung, prostate, colon, and pancreatic cancers present with cachexia (Tisdale, 2009; Von Haehling et al., 2010; Fearon et al., 2011; Johns et al., 2013). Although studies are ongoing, we currently know that malignant tumors alter their surrounding environment via tumor-derived and host-derived paracrine factors, which promote cachexia and may also lead to mitochondrial dysfunction. Some recent studies found evidence of mitochondrial dysfunction in cachexia, which may contribute to muscle pathology (Tan and Fearon, 2008; Constantinou et al., 2011; Fearon et al., 2011; Julienne et al., 2012, 2014; Wang et al., 2012; White et al., 2012; Dumas et al., 2013; Fontes-Oliveira et al., 2013; Tzika et al., 2013).

Mitochondria are multi-functional organelles that provide a majority of ATP to cells, are the major source of reactive oxygen species (ROS), and participate in multiple signaling cascades, including apoptosis. Reactive oxygen species play an important role in maintenance and adaptation of skeletal muscle (Irrcher et al., 2009; Merry et al., 2010; Dutka et al., 2012; Luo et al., 2013; Michaelson et al., 2013; Prosser et al., 2013). At low concentrations, ROS function in homeostatic signaling cascades, while at high concentrations they cause damage by oxidizing DNA, proteins, and lipids (Lee et al., 2012); in skeletal muscle, excessive oxidant production affects skeletal muscle mass and function in ways consistent with cachexia (Andrade et al., 1998, 2001; Reid et al., 2005; Hardin et al., 2008; Gilliam et al., 2011).

In this study, we examined the effect of Lewis lung carcinoma condition media (LCM) on mitochondrial function, protein content, and ROS production in C2C12 skeletal muscle myotubes. C2C12s are an immortalized cell line of mouse skeletal muscle that fuse and differentiate into myotubes under low-serum conditions (Yaffe and Saxel, 1977). They are a useful model is studying signaling pathways in skeletal muscle under controlled conditions. We chose Lewis lung carcinoma as our cancer model because it is known to induce cachexia and because lung cancer accounts for 23% of all cancer deaths worldwide (Carbo et al., 2004; Argiles et al., 2008; Jemal et al., 2011; Puppa et al., 2014). Then, we exposed C2C12 myotubes to LCM for 30 min, 2, 4, 24, or 48 h. We hypothesized that LCM would impair mitochondrial respiration, increase ROS production, and lead to oxidative stress. To test our hypothesis, we assessed the following in myotubes: mitochondrial oxygen consumption, content of mitochondrial complexes, voltage-dependent anion channel (VDAC), 4-hydroxynonenal (4HNE), and uncoupling protein 3 (UCP3), the activity of cytochrome c oxidase, and oxidant production via DCF, DAF, and Mitosox.

## Materials and methods

### Myotubes

C2C12 myoblasts (American Type Culture Collection) were plated at a density of 10,000 cells/cm^2^ in growth medium [Dulbecco's modified Eagle's medium (DMEM) with 10% fetal bovine serum, 1.6 g/L sodium bicarbonate, and 100U/ml PenStrep (Invitrogen)] and cultured at 37°C in 5% CO_2_. Cells reached ~90% confluence after 3 days, at which time cells were serum restricted in differentiation media (DMEM as above with 2% horse serum replacing fetal bovine serum). After 4 days of serum restriction, multinucleated myotubes were ready for treatment. Fresh medium was added every 2 days (Moylan et al., 2014).

### Lung cancer cells

Lewis lung carcinoma cancer cells (LL/2: American Type Culture Collection) were seeded at a density of 6000/cm^2^ in 100 mm cell culture plates in growth medium, as above. After 2 days, additional growth media was added to each plate. LL/2 cells are a heterogeneous mix of floating and adherent cells. After 4 days, we removed growth medium and harvested floating cells by centrifugation at 500 × g, 5 min. Pelleted cells and 10 mL differentiation media were added back to the adherent cells. After 2 days, we collected conditioned medium, removed cells and debris by centrifugation (500 × g, 5 min), and froze aliquots in liquid nitrogen for later use. For treatments, we used a 1 in 4 dilution of conditioned media mixed with fresh differentiation media.

### Western blot

C2C12 myotubes were homogenized in 2X protein loading buffer (120 mM Tris pH 7.5, 4% SDS, 200 mM DTT, 20% glycerol, 0.002% bromphenol blue). We separated proteins in equal volumes of lysates by SDS-PAGE (4–15% Criterion, BioRad) and determined relative total protein by scanning (Odyssey Infrared Imaging, LI-COR) stained gels (Simply Blue, Invitrogen). Fluorescence intensity data were used to normalize total protein for equal loading. Equal amounts of protein were separated by SDS-PAGE and transferred to PVDF membranes for western blot using the Odyssey System (Moylan et al., 2014). For preparation of mitochondrial and cytosolic fractions, mitochondria were isolated as in Lemire et al., and western blots were performed as above (Lemire et al., 2008).

### Antibodies

Primary antibodies were rabbit anti-VDAC and rabbit anti-4 HNE (Abcam), mouse anti-Complex I NDUFA9, mouse anti-Complex III, subunit core I, mouse anti-Complex IV, subunit IV (Novex), and mouse anti-ATP synthase α (Mitosciences). Secondary antibodies included anti-mouse IRDye 800CW and anti-rabbit IRDye 800CW (LI-COR).

### Oxidant assays and antioxidant treatments

To measure cytosolic oxidant activity, we aspirated off the cell culture media, replaced it with pre-warmed PBS supplemented with glucose (D-PBS) and 2′,7′-dichlorofluorescein diacetate (DCFH-DA 10 μM, Molecular Probes). We incubated C2C12 myotubes in DCFH-DA for 30 min, 37°C, 5% CO_2_. After incubation, we washed the cells twice with D-PBS, and added fresh D-PBS. To measure nitric oxide, we followed the same procedure as above using DAF-FM diacetate (4-amino-5-methylamino-2′,7′-difluorofluorescein diacetate) (DAF-FM 5 μM, Molecular Probes). We used a spectrofluorometer (Synergy H1, BioTek) to measure fluorescence of the oxidized derivatives, DCF (excitation: 480 nm, emission 520 nm) and DAF-FM (excitation: 495 nm, emission 515 nm), respectively. Antioxidant treatments included either 100 μM SS31, a mitochondrial-targeted antioxidant, or 5 mmol N-Acetylcysteine, a general antioxidant (Cho et al., 2007; Whiteman et al., 2008; Manczak et al., 2010; Powers et al., 2011). Both were administered simultaneously with LCM treatment.

### Fluorescence microscopy

We incubated C2C12 myotubes with MitoSox (5 μM, Molecular Probes), a mitochondrial superoxide anion specific indicator, for 30 min. Cells were rinsed twice with pre-warmed 1X PBS supplemented with glucose and sodium pyruvate. We captured live cell fluorescence images (excitation: 510 nm, emission 580 nm) using a CCD camera (CoolSNAP-ES, Roper Scientific Photometrics) attached to a Nikon TE2000 microscope with NIS Elements image acquisition software (Nikon).

### Oxygen consumption rate

We cultured C2C12 myotubes as above in XFe24 Analyzer plates (Seahorse Bioscience). Before running the assay, we replaced differentiation media with Seahorse assay medium supplemented with 10 mM sodium pyruvate and 10 mM glucose, and we adjusted the pH to 7.4. Seahorse assay medium is non-buffered for measuring proton production. Plates were equilibrated in this medium for 1 h at 37°C with no CO_2_. We used the same media as vehicle for our injection compounds. The analyzer sequentially injected the following compounds: oligomycin (1 μM final concentration), which blocks ATP synthase and shows the OCR dedicated to ATP production; carbonyl cyanide 4-(trifluoromethoxy) phenylhydrazone (FCCP: 4 μM final concentration), which permeabilizes the inner mitochondrial membrane and shows maximal OCR; and rotenone (1 μM final concentration), which blocks complex I and shows NADH-driven OCR. There were 3–4 wells per treatment group, and 4 plates in total. We measured OCR at three time points during basal respiration and after each injection using a Seahorse XFe24 Flux Analyzer (Seahorse Biosciences). We normalized OCR measurements to VDAC content in each well determined via western blot.

### Cytochrome C oxidase activity

We measured mitochondrial complex IV activity using a Cytochrome Oxidase assay kit (Biovision) per manufacturer's protocol. Briefly, we lysed C2C12 myotubes by 3 freeze-thaw cycles in 20 mM hypotonic potassium phosphate buffer pH 7.5 supplemented with protease and phosphatase inhibitor cocktails (Sigma). In a 96 well plate, we added Cytochrome c and Cytochrome Oxidase Assay Buffer (Biovision) to 2 μg total protein and recorded the decrease in optical density (OD) at 550 nm over 45 min using a spectrophotometer (Synergy H1, BioTek). The decrease in optical density is proportional to an increase in oxidized cytochrome c. We derived activity from the reaction rate in the linear phase, i.e., cytochrome oxidase activity (Units/mg) = [(OD_1_-OD_2_)/(t_1_-t_2_)]/(ε x mg protein), where ε is the molar extinction coefficient of cytochrome c at 550 nm (7.04 mM^−1^cm^−1^) and one unit oxidizes one μmole reduced cytochrome c per minute.

### Statistics

Prism 5.0b (GraphPad Software) and SAS served as our statistics software. We used *t*-test, One-Way ANOVA and *post-hoc* Bonferroni test, or repeated measures ANOVA as appropriate. Data are presented as mean ± SE. Statistical significance was defined as *p* < 0.05.

## Results

### Pre-treatment with LCM decreases mitochondrial respiration

**Figure 1:**
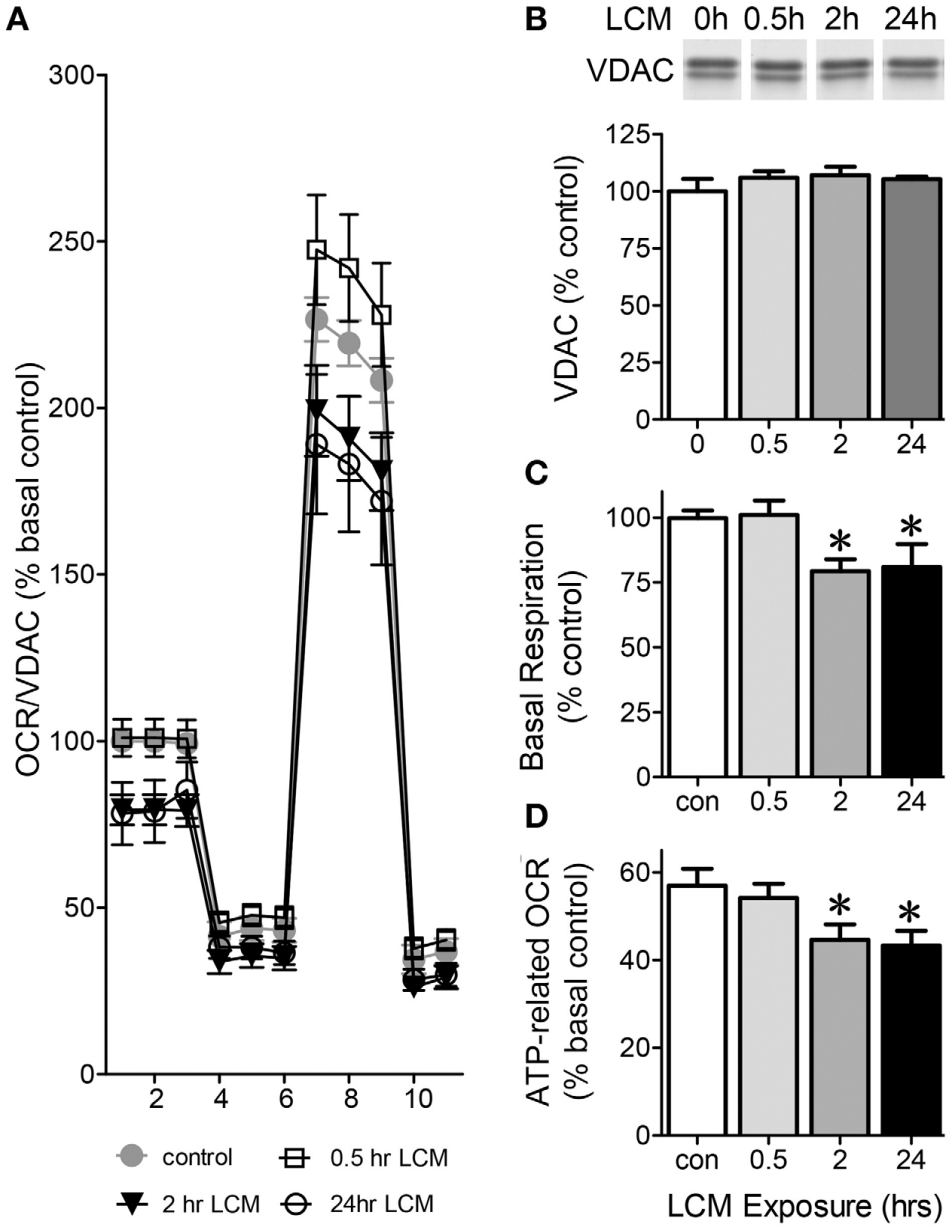
**Oxygen consumption rate (OCR) in C2C12 myotubes**. Eleven total OCR measurements were taken over 2 h: 3 basal respiration, 3 Oligomycin-sensitive respiration, 3 maximal respiratory capacity, and 2 non-mitochondrial respiration. The x-axis in **(A)** describes the measurement number. **(A)** OCR normalized to VDAC content presented at % basal control in control myotubes vs. myotubes treated for 30 min, 2 or 24 h with LCM (*n* = 12, ^*^P < 0.01 for treatment effect, repeated measures ANOVA). **(B)** VDAC content in myotubes treated with LCM, represented as percent control. **(C)** Change basal respiration in myotubes treated with LCM, expressed as percent of control respiration (*n* = 12, ^*^P < 0.01, ANOVA, Bonferroni *post-hoc*). **(D)** ATP-related OCR/VDAC in myotubes treated with LCM expressed as percent basal control (*n* = 12, ^*^P < 0.05, ANOVA, Bonferroni *post-hoc* test).

To assess how LCM affects mitochondrial function in skeletal muscle, we examined C2C12 myotubes after 30 min, 2, or 24 h in LCM. LCM treatment altered respiration in a time-dependent manner (Figure 1A). Western blots showed that VDAC content is unaffected by LCM treatment; therefore, we normalized OCR to VDAC content (Figure 1B). Thirty minutes of LCM treatment did not affect electron transport chain (ETC) activity; however, 2 or 24 h of LCM treatment resulted in depressed basal respiration (Figure 1C). The difference between basal respiration and oligomycin-sensitive respiration represents the OCR dedicated to ATP production. Compared to control myotubes, myotubes exposed to 2 or 24 h of LCM had significantly less ATP-related OCR (Figure 1D).

### Protein content of mitochondrial complexes in C2C12 myotubes treated with LCM

**Figure 2:**
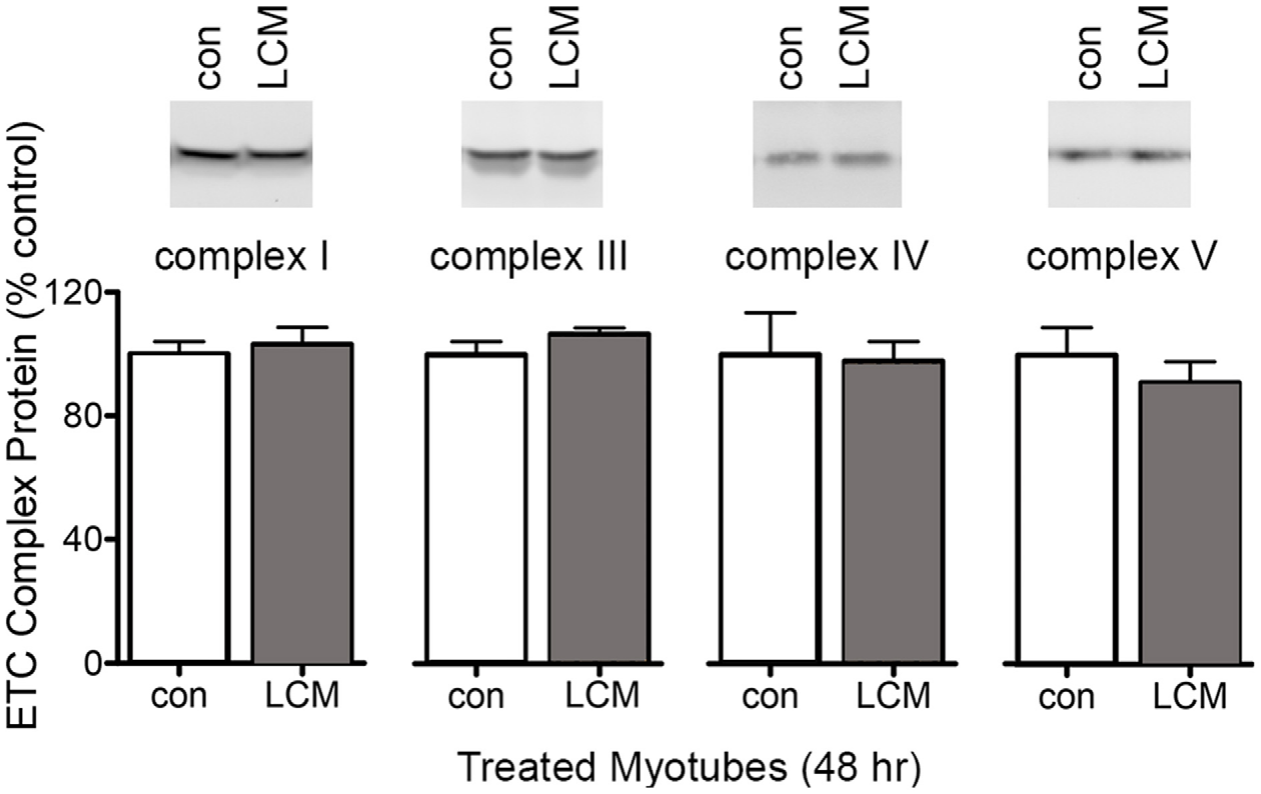
**Protein content of electron transport chain complexes**. Samples were equal protein loaded, which was determined via SDS-PAGE and staining with Simply Blue. Western blot membranes showed no changes in protein content for mitochondrial complex I NDUFA9, complex III subunit core I, complex IV subunit IV, and complex V, subunit α (*n* = 6).

To test whether the decreased mitochondrial respiration in LCM-treated myotubes was caused by changes in ETC complexes, we measured their content by western blot. Each complex in the ETC has multiple subunits, and we measured a representative subunit for each complex as follows: mitochondrial complex I NDUFA9; complex III subunit core I; complex IV subunit IV; and complex V, subunit α. LCM treatment did not alter the content of any ETC complex subunit measured (Figure 2).

### Effect of LCM treatment on cytochrome C oxidase activity

**Figure 3:**
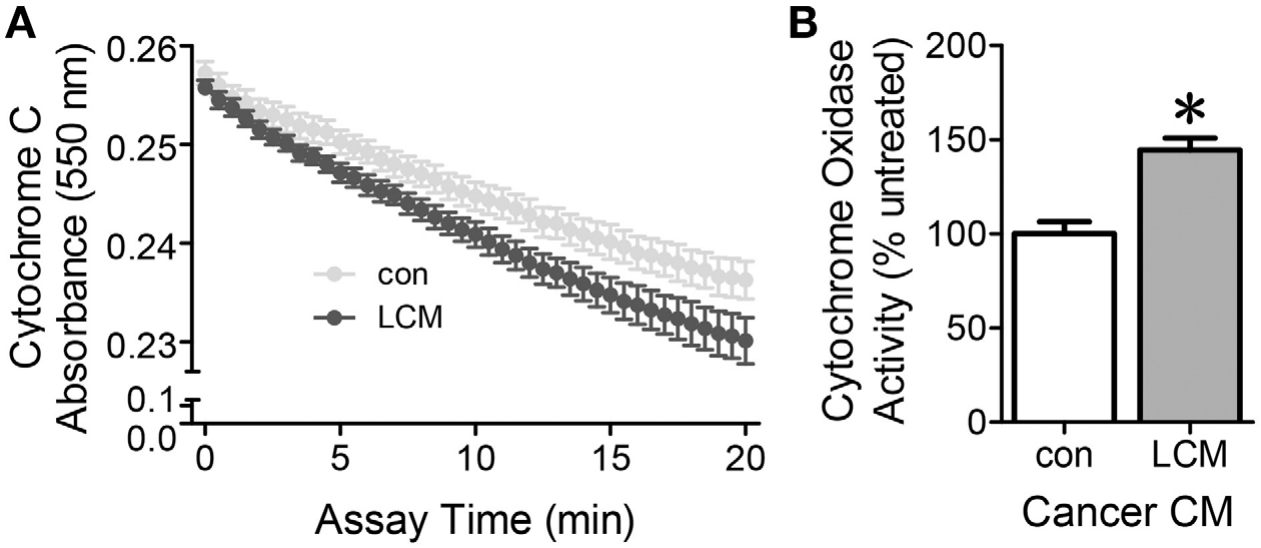
**The effect of LCM treatment on cytochrome c oxidase activity**. **(A)** Cytochrome C absorbance decreases after a 2 h exposure to LCM, indicating an increase in cytochrome c oxidase activity. **(B)** Cytochrome oxidase activity defined by the rate of change in the linear range per mg protein. LCM treatment for 2 h increased activity to 144% of control (*n* = 8, ^*^P < 0.01, *t*-test).

Cytochrome c oxidase is the last complex in the electron transport chain. It transfers four electrons to molecular oxygen. A decrease in cytochrome c absorbance indicates an increase in cytochrome c oxidase activity. Cytochrome c oxidase activity in myotubes treated with LCM for 2 h increased compared to control myotubes (Figure 3). These results suggest that there is an early burst of cytochrome c oxidase activity upon exposure to LCM.

### LCM-induced changes in oxidant production

**Figure 4:**
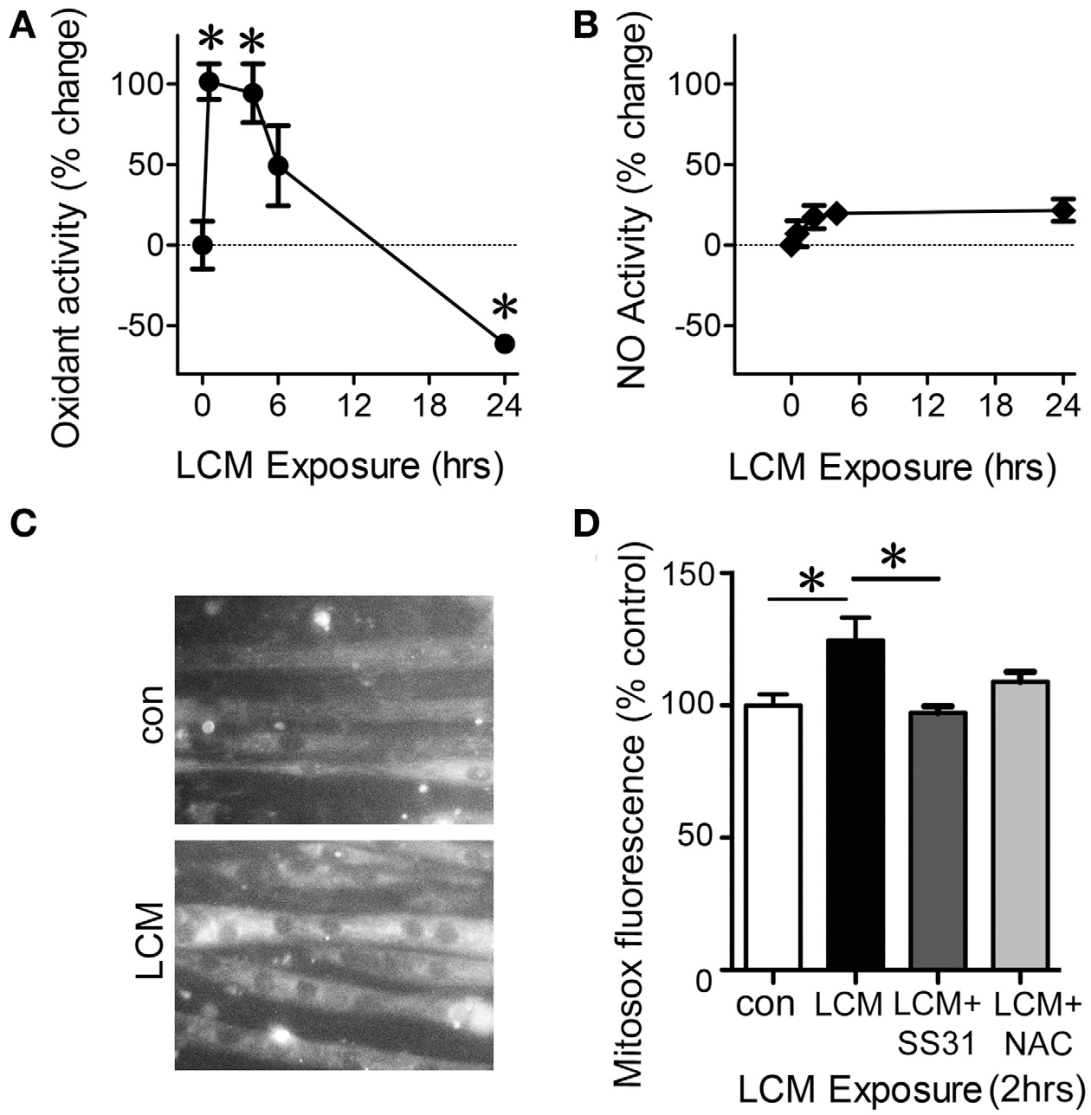
**LCM treatment alters oxidant production**. **(A)** DCFH fluorescence, a measurement of cytosolic oxidant levels, was increased by LCM treatment for 30 min and 4 h, but reduced by 24 h of treatment, represented as percent change (*n* = 10, ^*^P < 0.05, ANOVA, Bonferroni *post-hoc*). **(B)** Unaltered DAF fluorescence in myotubes treated with LCM for 30 min, 2, and 24 h, represented as percent change (*n* = 20, non-significant, ANOVA, Bonferroni *post-hoc*). **(C)** Fluorescence images of control myotubes (top panel) and LCM-treated myotubes (bottom panel) treated with MitoSox. **(D)** 2 h of LCM treatment increased MitoSox fluorescence compared to control; 2 h of LCM treatment combined with SS31 decreased Mitosox fluorescence compared to LCM-treated myotubes; and 2 h of LCM treatment combined with NAC did not alter MitoSox fluorescence significantly (*n* = 4, ^*^P < 0.05, Bonferroni *post-hoc*).

The ETC constitutively produces reactive oxygen species (ROS) during activity and is the greatest source of oxidants in skeletal muscle (Turrens, 2003). It is therefore likely that changes in ETC activity are reflected in alterations in ROS production. To test our hypothesis that LCM treatment would alter the level of oxidants, we examined general oxidant production, reactive nitrogen species (RNS) production, and mitochondrial superoxide anion production in myotubes treated with LCM for 30 min, 4, 6, and 24 h. DCF fluorescence measures overall cytosolic oxidant activity. Cytosolic oxidants increased at 30 min and 4 h compared to control myotubes (Figure 4A). However, after 24 h of LCM treatment, cytosolic oxidants decreased (Figure 4A). DAF fluorescence, which measures RNS production, was unaltered by LCM treatments (Figure 4B). These data indicated that RNS did not contribute to changes in overall cytosolic oxidant activity, as measured by DCF fluorescence. We hypothesized that some of the changes in DCF fluorescence might originate from the ETC. Using a CCD camera attached to a fluorescence microscope, we tested our hypothesis by measuring changes to the fluorescence intensity of mitochondrial superoxide indicator, MitoSox, in myotubes treated with LCM, LCM combined with mitochondrial-targeted antioxidant, SS31, and LCM combined with general antioxidant, N-Acetylcysteine (NAC). We found that after 2 h of LCM treatment, MitoSox fluorescence increased compared to control myotubes (Figures 4C,D). Myotubes treated for 2 h with LCM combined with mitochondrial antioxidant, SS31, had reduced MitoSox fluorescence compared to myotubes treated with LCM alone (Figure 4D). Myotubes treated with LCM combined with NAC had no significant alteration in MitoSox fluorescence (Figure 4D).

### The effect of LCM treatment on UCP3 content

**Figure 5:**
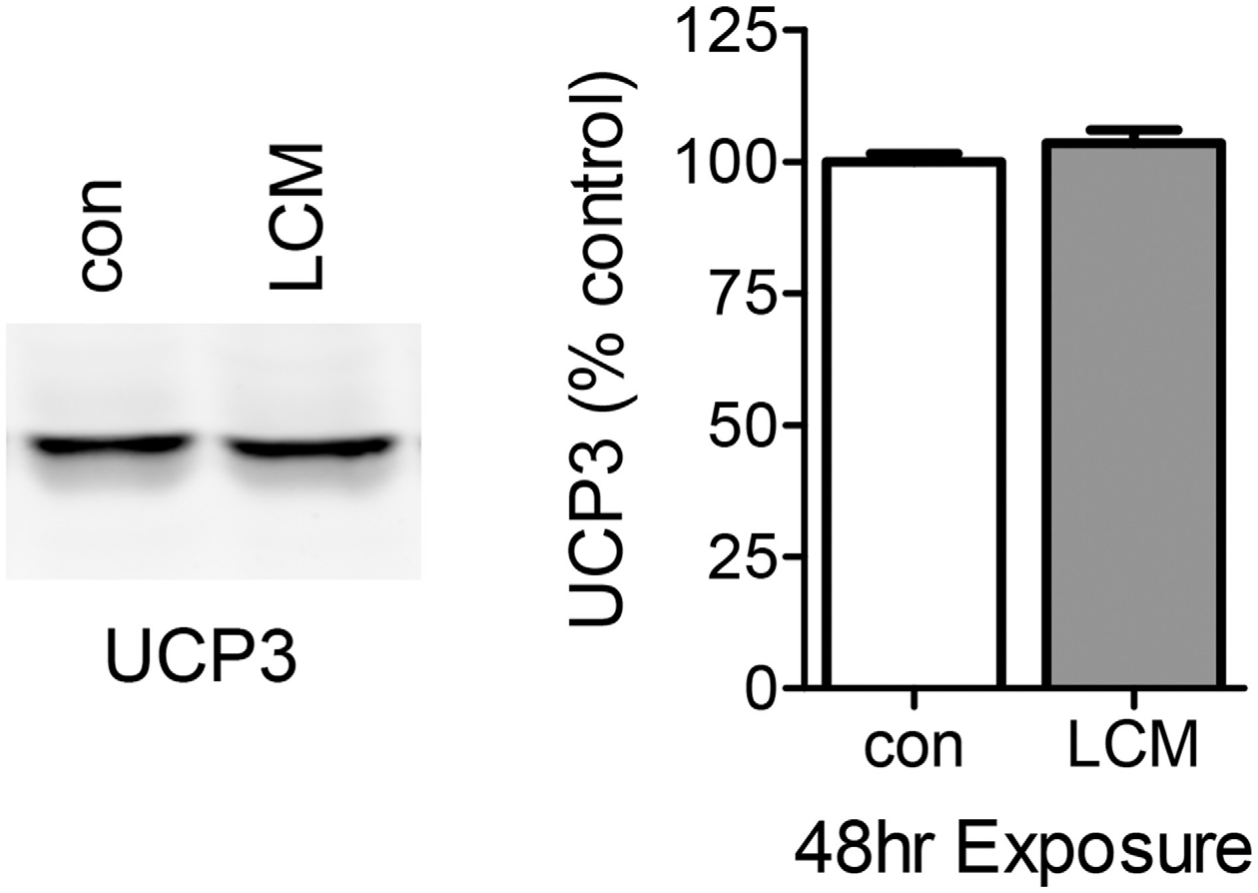
**The effect of LCM treatment on UCP3 content**. The left panel shows a representative western blot with anti-UCP3 in control and LCM-treated myotubes. The right panel shows the quantification of western blot represented as percent control (*n* = 6, *t*-test). 24 h of LCM treatment does not alter UCP3 content.

The DCF results with 24 h of LCM treatment are consistent with ETC uncoupling. UCP3 is the most abundant uncoupling protein in skeletal muscle, and we speculated that its expression may be elevated with prolonged LCM exposure as a protection against LCM-induced oxidative damage by reducing ROS production (Maclellan et al., 2005; Toime and Brand, 2010). We examined the content of UCP3 via western blot and found that its expression was unaffected by LCM treatment (Figure 5).

### Effect of LCM treatment on protein oxidation

**Figure 6:**
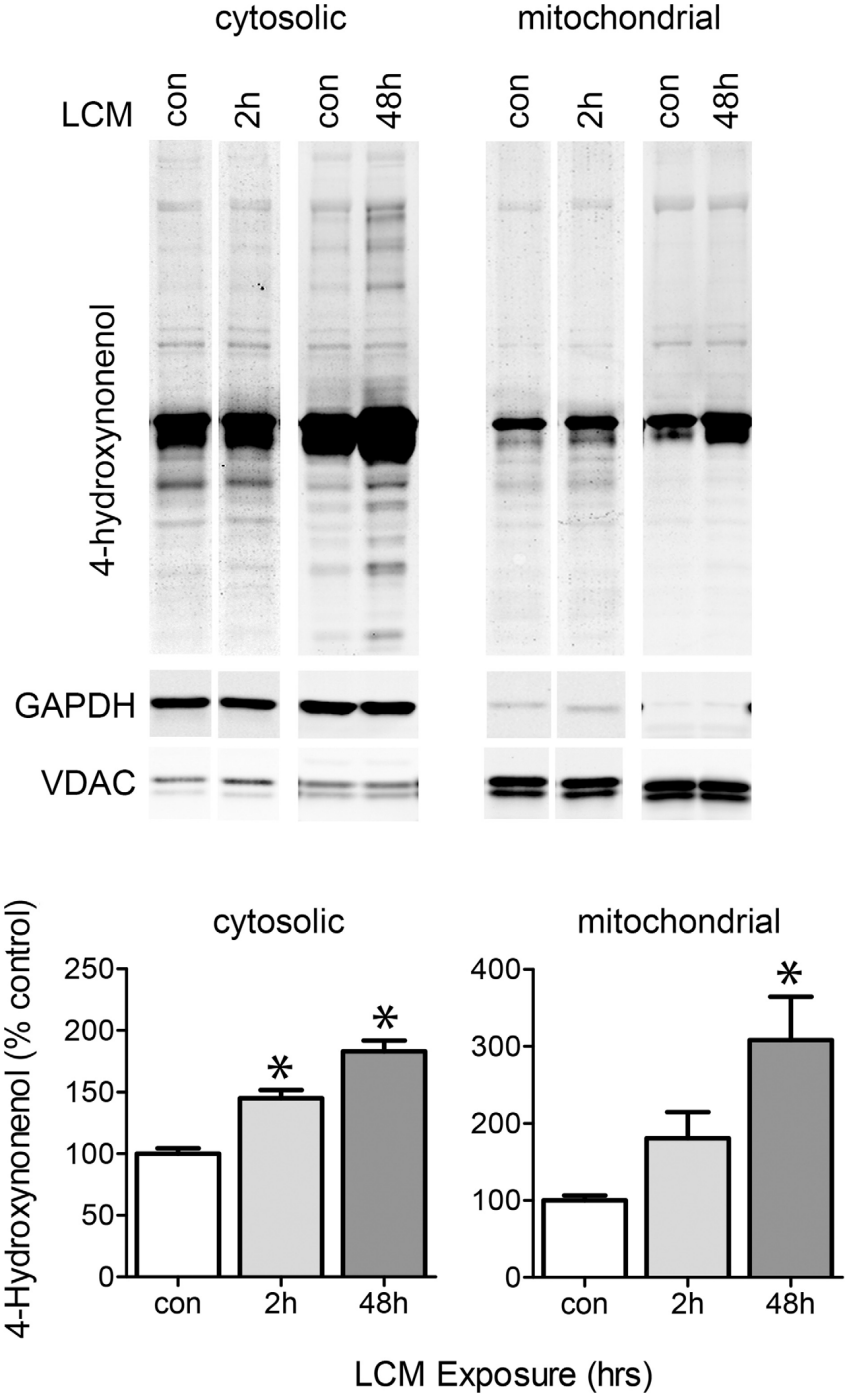
**LCM treatment oxidizes proteins**. The upper panel shows western blot for anti-4HNE in cytosolic and mitochondrial myotube fractions treated with LCM for 2 or 48 h, vs. control myotubes. The bottom panel shows the quantification anti-4HNE western blots (*n* = 4, ^*^P < 0.05, ANOVA, Bonferroni *post-hoc*). LCM treatment for 2 and 48 h increased HNE content in both cytosolic and mitochondrial portions of cell lysates.

LCM treatment caused a transient increase in ROS, potentially leading to oxidized proteins that may affect ETC activity (Figure 4). We isolated mitochondria from myotubes treated for 2 or 48 h with LCM. After 2 h of LCM treatment, 4-hydroxynonenal (HNE) was elevated in the cytosolic fraction, and after 48 h, HNE was elevated in both cytosolic and mitochondrial fractions (Figure 6). These data indicate that LCM oxidizes both cytosolic and mitochondrial proteins, which may result in alterations to their functions.

## Discussion

Mitochondrial dysfunction has been reported in several models of cancer cachexia; however, there have been conflicting data regarding the nature of alterations to the ETC (Busquets et al., 2005; Constantinou et al., 2011; Julienne et al., 2012; Khamoui and Kim, 2012; Tzika et al., 2013). One primary feature of cachexia is that the remaining muscle mass becomes easily fatigued, suggesting possible ETC dysfunction. For example, alterations to the ETC could promote fatigue through loss of ATP production, increased production of ROS, or both (Roberts et al., 2013a,b). Previous studies have found that excessive ROS oxidize myofibrillar proteins in skeletal muscle, and it is reasonable to hypothesize that ROS may also contribute to cachectic fatigue in this manner (Bruton et al., 2008; Reid, 2008; Prochniewicz et al., 2008a,b; Fedorova et al., 2010). In this study, we used an *in vitro* model of cancer cachexia to focus on the direct action of cancer-conditioned media on mitochondrial function, protein content, ROS production, and oxidative stress in skeletal muscle.

Our results show that LCM changed ETC activity in C2C12 myotubes in a time-dependent manner (Figure 1A). ETC activity was altered by LCM treatment. LCM exposure of 2 and 24 h lead to depressed basal respiration and ATP-related OCR (Figures 1C,D). The altered ETC activity with longer treatment times could have several possible explanations: less mitochondria in LCM-treated cells, reduced content of mitochondrial complexes, or increased ROS production. We tested protein content of VDAC as a measurement of mitochondrial content and found it unaffected by LCM treatment (Figure 1B). With this finding, we used VDAC as a means of normalization in XF analyzer measurements of OCR. We also measured the protein content of ETC complexes I, III, IV, and V, and found that LCM treatment did not alter expression (Figure 2). With content of mitochondria and the complexes remaining intact, alterations to complex activity became a candidate to explain our OCR results. Complex IV, cytochrome c oxidase, is the last electron carrier of the ETC, passing four electrons from reduced cytochrome c to molecular oxygen to produce water. If complex IV activity is diminished, it suggests that electron transport is slowed. We found that 2 h of LCM treatment increased Complex IV activity, which seems to contradict our hypothesis (Figure 3). However, Turrens found that ETC-generated superoxide can reduce cytochrome c directly leading to increased Complex IV activity while bypassing complexes I and III (Turrens, 2003). In other words, the activity could increase if more reduced cytochrome c substrate was present due to an increase in superoxide production.

The complex IV activity data suggested the possibility that LCM treatment caused an increase in ROS production. Although normal physiological activity of the ETC constitutively produces some ROS as electrons slip off of complexes I and III, excessive oxidant production is known to cause oxidation damage to proteins, lipids, and DNA (St-Pierre et al., 2002; Turrens, 2003). We hypothesized that LCM treatment was inducing excessive oxidant production, leading to oxidative stress in our model. LCM treatment initially increased oxidant production, but by 24 h after treatment, oxidants were depressed below control conditions (Figure 4A). Lack of changes in DAF fluorescence eliminated RNS contributions in our system (Figure 4B). Increased MitoSox fluorescence with LCM treatment implicated mitochondrial superoxide as a source for the initial rise in oxidant production (Figure 4C). This hypothesis was supported by additional experiments in which we showed a decrease in MitoSox fluorescence when LCM treatment was combined with mitochondrial targeting antioxidant, SS31, but not with general antioxidant, NAC (Figures 4C,D) An increase in superoxide may also explain our increase in cytochrome c oxidase activity.

Depressed oxidant production after 24 h of LCM treatment suggests that uncoupling may occur in our model. Uncoupling increases in response to elevated ROS production as a means of reducing ROS and the potential damage they inflict (Maclellan et al., 2005; Toime and Brand, 2010). Mitochondria are considered uncoupled when electrons move through the inner mitochondrial membrane without participating in ATP production. Other studies on cachectic skeletal muscle have found both increased and unchanged expression of uncoupling protein three (UCP3), the main uncoupling protein in skeletal muscle (Vidal-Puig et al., 1997; Constantinou et al., 2011; Julienne et al., 2012; Antunes et al., 2014). We found no change in the expression of UCP3 despite the depression in ROS production (Figure 5). With UCP3 unchanged, we wondered if oxidative damage might affect ETC activity, leading to a subsequent decrease in ETC-generated ROS production over time.

We tested the effect of LCM treatment on oxidative stress. 4-hydroxynonenal is a product of lipid peroxidation, and acts as both a marker and inducer of oxidative stress. It is known to aggregate and cause oxidative damage to DNA and proteins (Chen and Niki, 2006). We tested for 4HNE-modified proteins in cytosolic and mitochondrial fractions of myotube lysates via anti-4HNE western blot. We found that LCM increased 4HNE modifications to proteins in both fractions after 48 h (Figure 6). These data show that LCM causes oxidative damage, perhaps affecting ETC activity.

## Conclusions

Our results show that skeletal muscle cells treated with LCM experience altered ETC activity, including reduced basal respiration and reduced ATP-related OCR. LCM treatment initially induces a burst of ROS production, some of which is mitochondrial superoxide. Our 4HNE results show that LCM induced oxidative damage. 4HNE itself can act as a mediator of oxidative stress, and potentially creates a self-amplifying loop of oxidative stress. Both ROS and 4HNE can damage DNA, proteins, transcription factors, and lipids, resulting in widespread cellular damage. Future work should focus on discovering which LCM-derived paracrine factors are the upstream mediators in mitochondrial ETC alterations seen in cachexia.

## Author contributions

Julie B. McLean and Jennifer S. Moylan performed experiments and analyzed data; Jennifer S. Moylan and Julie B. McLean prepared figures, Julie B. McLean drafted manuscript, Julie B. McLean, Jennifer S. Moylan and Francisco H. Andrade edited and revised manuscript; Julie B. McLean, Jennifer S. Moylan and Francisco H. Andrade conceived experimental design; Julie B. McLean, Jennifer S. Moylan and Francisco H. Andrade approved final draft.

### Conflict of interest statement

The authors declare that the research was conducted in the absence of any commercial or financial relationships that could be construed as a potential conflict of interest.

## Abbreviations

ETC: electron transport chain
LCM: Lewis lung carcinoma conditioned medium
OCR: oxygen consumption rate
PAGE: polyacrylamide gel electrophoresis
VDAC: voltage dependent anion channel.
